# Tidally disrupted stars as a possible origin of both cosmic rays and neutrinos at the highest energies

**DOI:** 10.1038/s41598-018-29022-4

**Published:** 2018-07-17

**Authors:** Daniel Biehl, Denise Boncioli, Cecilia Lunardini, Walter Winter

**Affiliations:** 1 Deutsches Elektronen-Synchrotron (DESY), Platanenallee 6, D-15738 Zeuthen, Germany; 2 Department of Physics, Arizona State University, 450 E. Tyler Mall, Tempe, AZ 85287-1504 USA

## Abstract

Tidal Disruption Events (TDEs) are processes where stars are torn apart by the strong gravitational force near to a massive or supermassive black hole. If a jet is launched in such a process, particle acceleration may take place in internal shocks. We demonstrate that jetted TDEs can simultaneously describe the observed neutrino and cosmic ray fluxes at the highest energies if stars with heavier compositions, such as carbon-oxygen white dwarfs, are tidally disrupted and these events are sufficiently abundant. We simulate the photo-hadronic interactions both in the TDE jet and in the propagation through the extragalactic space and we show that the simultaneous description of Ultra-High Energy Cosmic Ray (UHECR) and PeV neutrino data implies that a nuclear cascade in the jet is developed by photo-hadronic interactions.

## Introduction

The discovery of high-energy (~0.1–1 PeV) astrophysical neutrinos^1^ has triggered substantial research on their possible origin. These neutrinos come probably from outside our galaxy, and can naturally arise from a flux of parent protons or nuclei. These facts together with basic energy budget considerations^2,3^ suggest that they may have the same origin as the Ultra-High Energy Cosmic Rays (UHECRs). Although many studies have been made to investigate a common origin of the IceCube neutrinos and the UHECRs (e.g.,)^4–9^, a class of astrophysical objects that is responsible for both UHECRs and neutrinos has not been identified. Models for the traditional candidates, such as Gamma-Ray Bursts (GRBs)^10^ and Blazars^11^, are disfavored to power the whole diffusive flux by neutrino stacking analyses, and this has stimulated research on alternative scenarios (see e.g.,^12,13^ for recent efforts).

Tidal Disruption Events (TDEs) are one such alternative. Tidal disruption is the process by which a star is torn apart by the strong gravitational force of a nearby massive or supermassive black hole. About half of the star’s debris remains bound to the black hole, and is ultimately accreted. It is predicted^14–17^ that TDEs with the highest mass accretion rate should generate a relativistic jet. This jet can accelerate protons or nuclei to ultra-high energies^18,19^, with neutrinos expected as a byproduct^20,21^. To date, three jet-hosting (“jetted”) TDEs have been robustly identified in X-rays observations^22–24^ (see also^25,26^), with the best observed one being Swift J1644 + 57^22^. Overall, they are consistent with a supermassive black hole (SMBH, $M > {10}^{5}{M}_{\odot }$) disrupting a main sequence star^22,27^. Intermediate mass black holes (IMBH, $M\sim {\mathrm{(10}}^{3}-{10}^{5}){M}_{\odot }$) are capable of disrupting smaller and denser stars (see e.g.,^28^), such as white dwarfs (WD), and indeed WD disruption is a viable alternative explanation of current data^29^. Regardless of the specific interpretation of observations, it is natural to expect diversity in the population of TDEs, involving black holes spanning many orders of magnitude in mass, as well as different types of stars.

TDEs as the sources of extragalactic neutrinos^20,30–33^ and UHECRs^28,34,35^ have been recently very actively discussed in the literature. Notably, refs^28,34^ focused on the recent observation of a mixed nuclear composition of UHECRs by the Pierre Auger Observatory^36^. They discussed how TDEs offer an attractive and natural explanation of the composition if the disrupted stars have mid-to-heavy compositions.

So far, a consistent study of the joint production of UHECRs and neutrinos in the jet generated by tidal disruption has not been performed. In a detailed discussion, Zhang *et al*.^34^ concluded that the most prominently used scenario, the internal shock model, generally faces the difficulty that nuclei will disintegrate *in the jet*, leading to a complex pattern of production of secondary nuclei and neutrinos. As a workaround, it has been assumed that the UHECRs come from regions with low enough radiation densities, where the disintegration rate (and also the neutrino production rate) is negligible.

In this work, we present the first consistent calculation of neutrino and UHECR production in TDE jets in the internal shock scenario. Our main purpose is to demonstrate that TDEs, with appropriate nuclear injection composition, are a viable common origin for the neutrinos and UHECRs, and to identify the relevant parameter ranges. The nuclear cascade in the source is modeled explicitly, using techniques that have been successfully applied before to GRBs^37,38^.

## Results

We model the TDE jet emission numerically, using values of the jet parameters that are consistent with the Swift J1644 + 57 observation^22^, as done in ref.^33^. For the sake of simplicity, we assume that a single nuclear species, ^14^N, is injected in the jet. This pure injection composition has been found to approximate the results obtained with a mixed carbon-oxygen (C-O) injection, which might be expected in the disruption of a C-O WD. This choice is also inspired by the recent observations of nitrogen emission lines in TDE observations^39,40^. Other possibilities for the nuclear composition, including ONeMg dwarfs from past supernovae or WDs with explosive nuclear burning (see *e.g*.), are other options which will not be considered here for brevity.

We simulate the interactions in the TDE jet with the *NeuCosmA* code as in^38^. The resulting cosmic ray and neutrino spectra are then processed by the *SimProp* code^41^, which models the UHECR propagation through the extragalactic space, and also computes the cosmogenic neutrino flux. The mechanism for the escape of the cosmic rays from the sources is calculated as in ref.^42^, leading to hard spectra ejected from the source and injected in the extragalactic space. These spectra are compatible with the results from the UHECR global fit by the Auger Collaboration^43^ (depending on the source evolution). We obtain the diffuse particle fluxes at Earth, using the assumption that all TDE jets are identical in the cosmologically co-moving frame, and that their rate evolves negatively with the redshift (approximately as $\sim {\mathrm{(1}+z)}^{-3}$), following the evolution of the number density of SMBHs as calculated in ref.^44^ (see also)^33,45,46^. We also compute the first two moments of the distributions of the quantity *X*_max_, which is defined as the depth at which the energy deposited in the atmosphere by a cosmic ray shower reaches its maximum; *X*_max_ depends strongly on the mass of the primary cosmic ray nucleus.

To assess the compatibility with observations, we analyze the Pierre Auger Observatory data for the UHECR spectrum^47^ and for the distributions of *X*_max_^36^ beyond ${10}^{19}{\rm{eV}}$. A fit of these data is performed, including a downshift (of the data) of 20% in the energy scale to better match the maximal energy of the spectrum. The shift amount is comparable to the energy scale uncertainty of the Auger experiment (14%, as reported in^48^). It is treated as experimental systematics here, but it is degenerate with the acceleration efficiency (or even nuclear injection composition) of the primaries, which can be adjusted accordingly to reach high enough maximal energies. As a consequence, due to the uncertainties on the source composition and the acceleration efficiency, the shift in the energy scale cannot be deduced from the current theoretical source model. After the UHECR fit, as a separate step, we check the compatibility of the results with the IceCube neutrino data (measured data points beyond PeV energies^49^).

The UHECR fit is performed using the maximum likelihood method, with three fit parameters: the production radius *R* (distance from black hole where internal shocks occur), the X-ray luminosity *L*_*X*_, and a single normalization parameter, *G*. The latter takes into account the degeneracy between the baryonic loading *ξ*_*A*_–defined as the energy injected as nuclei over the total X-ray energy in the Swift range 0.4–13.5 keV^22^ – and the local apparent rate of jetted TDEs $\tilde{R}\mathrm{(0)}$. It is defined as

1 $$G\equiv {\xi }_{A}\times \frac{\tilde{R}\mathrm{(0)}}{0.1\,{{\rm{Gpc}}}^{-{\rm{3}}}\,{{\rm{yr}}}^{-{\rm{1}}}}\mathrm{.}$$

The reference value chosen for $\tilde{R}\mathrm{(0)}$ is the rate of WD-IMBH disruptions inferred from observations^32,34^ $\tilde{R}\mathrm{(0)}\sim 0.01-0.1\,{{\rm{Gpc}}}^{-{\rm{3}}}\,{{\rm{yr}}}^{-{\rm{1}}}$ (which is in agreement with theoretical arguments, see *e.g*.^29^).

**Figure 1:**
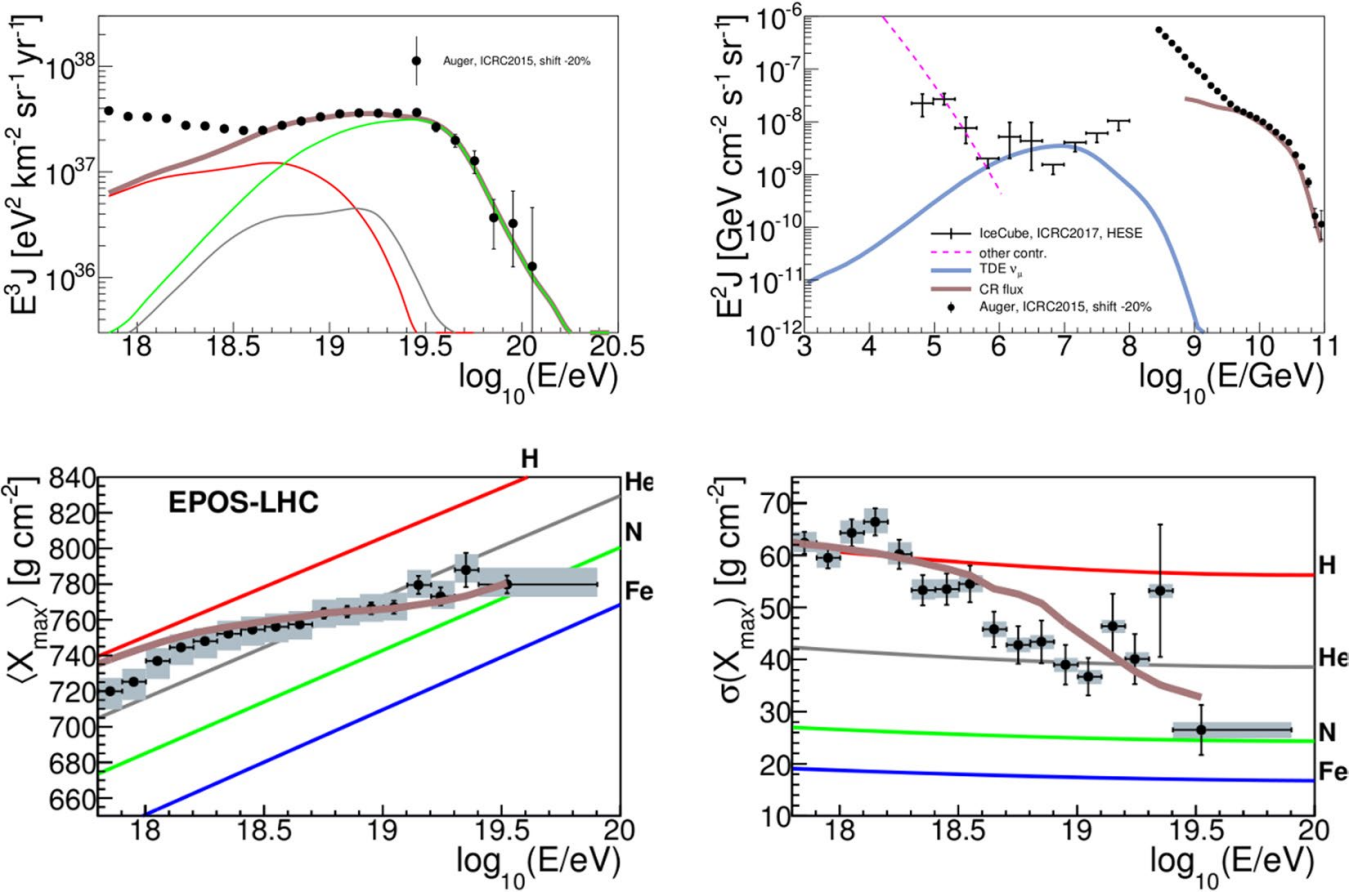
Cosmic ray and neutrino observables corresponding to a parameter space point describing both UHECR and neutrino data at the highest energies (point A in Fig. 2, *L*_*X*_ = 10^47^ erg/s, *R* = 10^9.6^ km, with *G* = 540). *Upper right panel*: Predicted muon neutrino spectrum from TDEs, compared to the data from the High Energy Starting Events at IceCube^49^. An additional flux, called ‘other contribution’ (see main text for details), is also shown. The simulated energy spectrum of UHECRs multiplied by *E*^2^ and the Auger spectrum data points^47^ are also shown. *Upper left panel*: Simulated energy spectrum of UHECRs (thick curve), multiplied by *E*^3^; and its components from (groups of) different nuclear species (thin, same color coding as in the bottom panels). For comparison, the Auger data points are shown^47^. *Lower panels*: Predictions and data^84^ on the average (left) and standard deviation (right) of the *X*_max_ distributions as a function of the energy. For predictions, EPOS-LHC^85^ is assumed as the interaction model for UHECR-air interactions. A shift of −20% is applied to the energy scale of all the UHECR data, see text.

Figure 1 shows our result for a parameter space point fitting UHECR (upper left panel and lower panels) and describing the PeV neutrino data (upper right panel). One can easily see that the UHECR spectrum and *X*_max_ beyond 10^18.7^eV, and the neutrino spectrum at PeV energies are reproduced very well. By showing the cosmic-ray flux multiplied by *E*^2^ in the upper left panel together with the neutrino flux it is also clear that the energy level of these fluxes is comparable.

The upper limit at 6–10 PeV is obtained by the non-observation of the Glashow resonance, assuming that the flux of neutrinos is equal to the flux of anti-neutrinos at Earth^1^. However, this does not hold for *pγ* interactions, which can significantly change the expected number of events^50,51^. For *pγ* sources, which could be even muon-damped (inhibited muon decay due to fast secondary cooling), the constraint can be relaxed by a factor up to ~4–5. By comparing the energy loss and decay time scales at the corresponding muon energy, which is about 3 times the neutrino energy, we estimate the magnetic field to be at least ~2 kG in order to efficiently cool muons in this case. Further observations will therefore constrain the source parameters^52^. The expected event number in IceCube for the flavor composition of our best fit flux is ~1.2 events in 6 years of operation, which is consistent with a non-detection within the uncertainties. However, IceCube recently observed a 5.9 PeV event, which might be compatible with a Glashow resonance event^53^.

The lower energy neutrino flux cannot be described with our model; the additional flux needed to reproduce the data in this region might originate from multiple components (see ref.^54^ for a detailed discussion on the evidence of multiple components), from choked jets accompanied by type II supernovae^55^ or decaying Dark Matter^56^, for example. We emphasize that, for the parameters in Fig. 1, the source is optically thick to photo-hadronic interactions at the highest energies. Therefore, the effect of nuclear disintegration in the source is important here. By including systematics (energy calibration error), we obtain a better fit compared to ref.^34^, where the UHECR data are described in the nuclear survival regime for the disruption of C-O WDs, and a poor fit to the energy spectrum is found.

**Figure 2:**
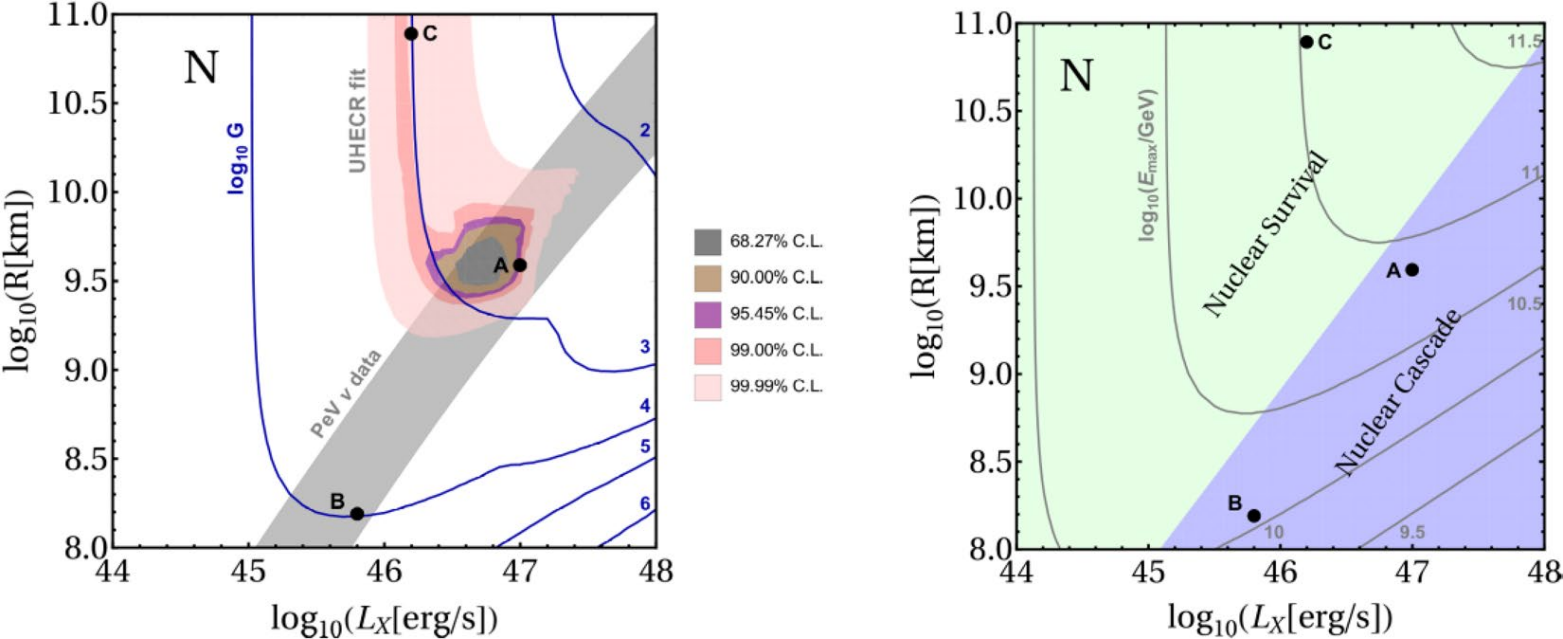
*Left panel*: Results of the fit to UHECR and the description of PeV neutrino data as a function of *L*_*X*_ and *R* (shaded contours, CL for two parameters). The curves show isocontours of ${\mathrm{log}}_{10}G$ (see Eq. (1)) obtained from the cosmic ray fit, where a shift of −20% is applied to the energy scale of all the UHECR data. For each point (*L*_*X*_,*R*), the value of *G* that maximizes the likelihood is used, *i.e*., *G* is marginalized in the fit. *Right panel*: Different regimes in the parameter space for the nuclear cascade to develop in the source (shaded regions), as discussed in the main text. The curves show ${\mathrm{log}}_{10}({E}_{max}/{\rm{G}}{\rm{e}}{\rm{V}})$, with *E*_max_ being the obtained maximal energy in the observer’s frame.

In Fig. 2, left panel, we show (filled) the confidence level contours for the fit to the UHECR data, in the space of *L*_*X*_ and *R*, after marginalizing over *G*; iso-contours of ${\mathrm{log}}_{10}G$ at the minimum are shown as well. We also superimpose the region where the predicted neutrino flux is within 1*σ* from the two PeV data points of IceCube, thus providing an acceptable description of them. Point A in the figure gives the parameters used in Fig. 1; point B marks the best description of the PeV neutrino data, and point C corresponds to a reasonable fit to the UHECRs in a different physics regime. For points B and C, only one data set (UHECR or neutrinos) can be described well, but not both. Note that for the UHECR data the statistical errors are smaller than the systematics ones; however, we find that the 99.99% CL region in Fig. 2 is wide enough to be representative of the fit results that can be obtained if systematics (such as on the cosmic ray propagation model and on the energy scale of the experiment, as discussed for example in^43,57^) are included (see also the Supplementary Material).

In order to understand what physics determines the allowed regions found in the fit, we show the different regimes of the nuclear cascade in the right panel of Fig. 2: the one where the collision region is optically thick to nuclear disintegration (“nuclear cascade”)^38^, and the complementary one where disintegration is inefficient (“nuclear survival”). Iso-contours of the maximal energy of the nuclei spectrum, *E*_max_, are shown as well. It appears that the allowed region of the UHECR fit mainly follows the contour ${E}_{max}\simeq {10}^{10.8}{\rm{G}}{\rm{e}}{\rm{V}}$ for the maximum energy in the source; this value indeed reproduces the UHE range observed of cosmic rays at at Earth. Instead, the region preferred by the PeV neutrino data correlates with the nuclear cascade region, because nuclear disintegration and neutrino production require similar (but not too high) radiation densities for the photo-nuclear processes. At point C, the UHECR spectrum and composition are reproduced but neutrino production is inefficient, thus resulting in a too low neutrino flux. At point B, the neutrino production is efficient enough to reach the level of the PeV data, but *E*_max_ is too low, which means that the high energy UHECR flux is not reproduced and the expected composition at Earth is heavier than what is measured. Note that by requiring efficient particle acceleration, a constraint on the radiation density can be obtained, as otherwise the shock may be radiation dominated^21^ (see also^58–60^). For fixed Lorentz factor Γ, this translates into a bound for the ratio of X-ray luminosity and collision radius. We find that ${L}_{X}/R\lesssim {10}^{41}$ erg s ^−1^ km ^−1^, which is consistent with the section of the parameter space shown here.

Our results can be physically interpreted in terms of different scenarios using the value $G\simeq 540$ obtained at point A. For WD disruptions, with the fiducial local rate $\tilde{R}\mathrm{(0)}\simeq 0.1\,{{\rm{Gpc}}}^{-{\rm{3}}}\,{{\rm{yr}}}^{-{\rm{1}}}$, this normalization is realized for a baryonic loading ${\xi }_{A}\sim 500$, see Eq. (1). Let us briefly estimate the naturalness of the energy budget. Taking ${M}_{{\rm{WD}}}\sim {M}_{\odot }$, and $\Gamma \sim 10$, one obtains the desired normalization, ${\xi }_{A}\sim 0.15\times 2{\Gamma }^{2}{M}_{{\rm{WD}}}{c}^{2}/{E}_{X}\simeq 525$, if 15% of the disrupted star’s mass is re-processed into non-thermal baryons in the jet (the factor 2Γ^2^ corrects for the beaming). For a jet duration of ${\rm{\Delta }}T\sim {10}^{6}$ s, the corresponding accretion rate is ${L}_{{\rm{a}}{\rm{c}}{\rm{c}}{\rm{r}}}\sim 0.15\times {M}_{{\rm{W}}{\rm{D}}}{c}^{2}/{\rm{\Delta }}T\simeq 1.3\times {10}^{47}{\rm{e}}{\rm{r}}{\rm{g}}\,{{\rm{s}}}^{-1}$. This rate can be compared to the Eddington luminosity of the black hole, ${L}_{{\rm{E}}{\rm{d}}{\rm{d}}}\sim 1.25\times {10}^{43}(M{/10}^{5}{M}_{\odot }){\rm{e}}{\rm{r}}{\rm{g}}\,{{\rm{s}}}^{-1}$, which is frequently used as a reference corresponding to the hydrostatic balance between gravitational and radiation pressure. The ratio ${L}_{{\rm{accr}}}/{L}_{{\rm{Edd}}}\sim {10}^{4}\times {(M{\mathrm{/10}}^{5}{M}_{\odot })}^{-1}$ is roughly consistent–considering the uncertainty on the Lorentz factor of the jet (larger Γ require smaller accretion rates)–with recent simulations^61^, which indicate that the accretion luminosity exceeds *L*_Edd_ by a factor $\sim {10}^{3}$ for $M\sim {10}^{5}\,{M}_{\odot }$.

The same value of the normalization *G* can be realized for a lower, more conservative, baryonic loading, at the expense of a higher local rate $\tilde{R}$(0). The requirement on the baryonic loading is also relaxed if one considers points in the upper right part of the allowed region of the parameter space (Fig. 2), where slightly lower values of *G* are obtained. Moreover, a lower baryonic loading is obtained for a spectral injection index from the acceleration harder than what we use in this work (*E*^−2^).

In addition to energy scale checks, a number of observational constraints must be considered. In particular, WD disruptions as dominant source of UHECRs or neutrinos are disfavored by generic considerations on X-ray sources as sources of the UHECRs^62^, and lower limits on the apparent local rate from the non-observation of neutrino multiplets (as indicators of point sources)^4,63,64^. Note, however, that we only have three (cascade) events in the energy range considered in Fig. 1, which means that the multiplet constraints are still loose due to the low event statistics^54^.

A potential limitation of our model is the assumed evolution of the TDE rate with redshift following the IMBH number density. Recent studies suggest that black holes of intermediate and small mass might be less numerous today than in the past because they may have merged into more massive black holes^65^. This would suggest a less negative, or even positive evolution of the TDE rate with redshift for which the combined description of UHECR and PeV neutrino data rapidly becomes more challenging (see also the Supplementary Material). Another limitation is that the input parameters assumed here have been kept fixed and inspired by the Swift J1644 + 57 observation. Alternative hypotheses include the option that ultra-long GRBs might be caused by the disruption of WDs^66,67^ (see, however,^68^), with GRB 111209 A being a candidate. Compared to Swift J1644 + 57, these bursts have a shorter duration and different X-ray spectra^69^ and possibly a shorter variability time scale^70^. Another option is the tidal disruption of neutron stars, which may be associated to gravitational wave events such as GW170817^71^. For example, the observed short GRB in the follow-up of this event^72^, SGRB 170817 A, may be interpreted as a representative of a new population of jetted TDEs then. More broadly, one may consider multiple classes of sources as contributing to the UHECR and neutrino fluxes.

## Discussion and Conclusions

In summary, we have demonstrated that TDE jets with mid-to-heavy nuclear composition can reproduce both the observed cosmic rays and neutrinos at the highest energies, with typical parameters ${L}_{X}\simeq {10}^{46}$ to ${10}^{47}{\rm{erg}}\,{{\rm{s}}}^{-{\rm{1}}}$, and $R\simeq {10}^{9.5}\,{\rm{km}}$ (distance of production region from black hole). We find that two important ingredients are necessary for a common description: the first is that nuclear disintegration should be efficient *in the jet*. This is because efficient neutrino production requires high radiation densities, which in turn implies efficient disintegration of nuclei. Therefore, the nuclear cascade in the jet has to be computed, and this computation is a key novelty of our work for TDEs. The second condition is that the evolution of the sources with redshift should be negative (*i.e*., the jets should be less frequent in the past than today). Indeed, this evolution is known to lead to a good fit of the UHECR data^73^, and also to preserve consistency of the neutrino flux^74^ with the measured extragalactic gamma-ray background^75^. Negative evolution is plausible for TDEs following the SMBH mass function, but debated for intermediate black hole masses. A consequence of it is that cosmogenic neutrinos will not be be detected, neither in the current nor in the next generation of experiments (see also the Supplementary Material). Energy budget considerations and the negative source evolution greatly restrict the classes of objects that can host such jets: for a large baryonic loading, a large mass ($M\sim 0.1-1{M}_{\odot }$) of material with heavy composition is required to be converted into the jet. Alternatively, a sufficiently large apparent source density is needed. Many sources (such as supernovae or the GRBs) which are expected to track the star formation rate, are excluded due to the source evolution argument. The disruptions of WDs by massive black holes may be an attractive option because of their carbon-oxygen composition and plausible rate and jet parameters.

This scenario is already somewhat in tension with upper limits on the jet baryonic loading, placed by the observed energy of Swift J1644 + 57, and lower bounds on the local rate of jets from searches of neutrino point sources. However, at this time it remains a viable possibility, in consideration of the large uncertainties on a number of astrophysical parameters, and especially the local rate of jetted TDEs from WD disruption.

Future observations and progress in theoretical modeling will help to substantiate, or disfavor, the TDE origin of UHECRs and neutrinos. The local rate of jetted TDEs may become better known through more advanced studies of the formation and evolution of massive and supermassive black holes. Work is especially needed to reduce the large uncertainties on the physics of IMBH; and pessimistic predictions on their present number density and on their galaxy occupation fraction (see *e.g*. ref.^76^) could disfavor the IMBH-WD disruption scenario we have described. Observationally, a higher number of precision observations of jetted TDEs will help to constrain their rate, and the jet parameters. An association of neutrinos with TDEs could be established from multi-messenger studies, by cross correlating–in time and position in the sky–the neutrino data with astronomical observations of TDEs. The latter may be available only at long wavelengths, such as the radio, infrared, and X-ray bands, while gamma-rays may not escape from high luminosity objects. A recent discovery^77^ supports this idea, as it indicates that some jetted TDEs may be obscured by dust, which means that not even the X-ray emission is visible. Note that for the chosen negative source evolution, the electromagnetic and neutrino observations may be indeed more indicative for the origin of the cosmic rays than for stronger source evolutions, because all messengers are dominated by close-by sources (see Supplementary Materials). Furthermore, the non-observation of neutrino point sources will limit the apparent rate. Finally, the recently observed short gamma-ray burst SGRB 170817 A associated with the gravitational wave event GW 170817 may be indicative of a new class of “tidal disruption events” (if interpreted as black hole-neutron star merger) which may be interpreted in a similar framework.

## Methods

The parameters chosen in this work are motivated by the observation of Swift J1644 + 57. Therefore, the target photon field is parameterized as a broken power law with a spectral break at *ε*_*X*,br_ = 1 keV in the observer’s frame and spectral indices *α* = −2/3 and *β* = −2 below and above the break energy, respectively, similar to the observed spectral energy distribution (SED). For this event, the isotropic equivalent luminosity of the X-ray flare was ${L}_{X}\simeq {10}^{47.5}$ erg s ^−1^ over a time of $\Delta T\simeq {10}^{6}$ s, leading to an estimated total energy of ${E}_{X}\simeq {L}_{X}\Delta T\simeq 3\times {10}^{53}$ erg. A Lorentz factor $\Gamma \simeq 10$ and a minimum variability time ${t}_{v}\simeq {10}^{2}$ s are estimated from the observations^22^. The collision radius, *i.e*., the distance of the shock from the central engine, is defined as *R* = 2Γ^2^*ct*_*v*_/(1 + *z*) in the internal shock model. When performing the fit, we keep Γ fixed and vary *R*, such that *t*_*v*_ is determined by the before mentioned relation.

We do not specify the acceleration mechanism, but we assume that cosmic rays gain energy by Fermi-like shock acceleration, i.e. the spectrum of primary injected nuclei follows $\propto \,{E}^{-2}{e}^{-E/{E}_{{\rm{\max }}}}$. Therein, *E*_max_ is obtained self-consistently by balancing the acceleration with energy loss processes and the dynamical time scale ${t}_{{\rm{dyn}}}\sim {\rm{\Delta }}d^{\prime} /c$, with the size of the region Δ*d*′. In the Supplementary Material the dependence of the fit results on the spectral index is reported. The acceleration rate is ${t^{\prime} }_{{\rm{acc}}}^{-1}=\eta c/{R^{\prime} }_{L}$, where *R*′_*L*_ = *E*′/*ZeB*′ is the Larmor radius of a particle with charge number *Z* and energy *E*′, and *η* is the acceleration efficiency. Charged particles can only escape if the edge of the shell is within their respective Larmor radius, i.e. over the dynamical time scale, a fraction *f*_esc_ = min(*R*′_*L*_(*E*),Δ*d*′)/Δ*d*′ ≤ 1 escapes. The magnetic energy density is assumed to be in equipartition with the photon energy density. We assume that acceleration is efficient, *i.e*., *η* = 1.0. Note that *E*_max_ (and therefore, indirectly, *η*) is somewhat degenerate with the systematic shift of the measured UHECR energy (see main text).

**Figure 3:**
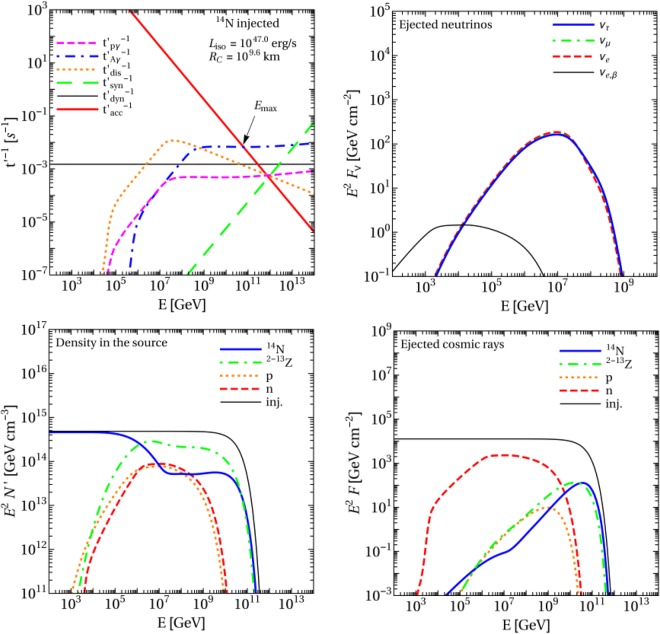
Interaction rates (upper left), neutrino fluence per flavor (upper right), isotope density in the source (lower left) and ejected cosmic ray fluence (lower right, no interactions in the propagation included) as a function of the energy in the observer’s frame at point A in Fig. 2 (*L*_*X*_ = 10^47.0^ erg s ^−1^ and *R* = 10^9.6^ km) for pure^14^ N injection. Ejected fluences take into account propagation with adiabatic energy losses only. The injected spectrum, called ‘inj’ is plotted in the lower left and lower right (propagated) panel for comparison. The other TDE parameters are chosen to be Γ = 10, *ξ*_*A*_ = 10, *ε*′_*γ*,br_ = 1 keV and *z* = 0.001.

When simulating the nuclear cascade, we distinguish between two energy (photon energy in the nuclei’s rest frame) regimes, the photo-disintegration (${\varepsilon }_{\gamma }\lesssim 150\,{\rm{MeV}}$) and photo-meson production (${\varepsilon }_{\gamma }\gtrsim 150\,{\rm{MeV}}$). The photo-meson production simulation is based on the SOPHIA code^78^. In it, a superposition model is used, meaning that for nuclei, the cross sections are approximated as scaling with the nucleus’ mass number, *A*, *i.e*., *σ*_*Aγ*_ = *Aσ*_*pγ*_ (with *σ*_*pγ*_ being the cross section for protons). The photo-disintegration uses the TALYS 1.8 code for nuclei with *A*≥12^79^ and CRPropa2 for lighter nuclei^80^ (for details see^37^). Figure 3 shows the interaction rates, the neutrino fluence, the isotope density in the source and the ejected cosmic ray fluence for the parameters of point A in Fig. 2. It appears that the maximum energy is limited by photo-meson production (${t^{\prime} }_{A\gamma }^{-1}$ exceeds ${t^{\prime} }_{{\rm{acc}}}^{-1}$ at ${E}_{{\rm{\max }}}\sim 6\times {10}^{10}$ GeV), implying that this is also the relevant process for disintegration at the highest energies. Note that in our superposition model for photo-meson production, which still corresponds to the state-of-the-art in the literature, a nucleon is assumed to interact with the photon and then leaves the nucleus. The interaction of the single nucleon is described with SOPHIA, whereas the remaining nucleus is assumed to stay intact. A more realistic model may involve additional disintegration of the remaining excited nucleus–which is to be studied in the future. One can also see that the photo-disintegration rate follows the low-energy photon spectral index above the break at about 10^7.5^ GeV (high-energy nuclei interact with low-energy photons), leading to a sub-dominant contribution at the highest energies (beyond about 10^8.5^ GeV) compared to the photo-meson production.

Note that we assume that the source is optically thick to high energy gamma ray escape, which is consistent with observations^22,81^. We discuss the implications of this in the Supplementary Material.

Figure 3 (upper right panel) shows the neutrino fluence in each flavor, in the observer’s frame, computed for a source at redshift *z* = 0.001. Neutrinos from beta decays (from isotopes within and outside the source) are only relevant at low energies, and are shown as a separate curve. The plot of particle densities inside the source (lower left panel) shows that the nitrogen spectrum is depleted–with respect to the *E*^−2^ injection spectrum–at the highest energies, where the isotopes produced in the disintegration chain dominate the spectrum. The spectrum of the ejected neutrons (given in Fig. 3, lower right panel) follows the spectrum within the source (lower left panel). Instead, the spectrum of the charged cosmic rays is harder (tilted in factor of higher energies), because we assume a direct UHECR escape mechanism (for details see^42^). This mechanism conservatively assumes that only particles from the boundaries of the production region can escape (within their Larmor radius, see above). Similar results are obtained for Bohm-like diffusion throughout the whole region. In this case, the escape is moderately efficient, as the Larmor radius is smaller than the size of the region at the highest energy (i.e. the acceleration is not limited by the dynamical time scale, see arrow in Fig. 3, upper left panel).

We also checked that the results in Fig. 3 are consistent with those in ref.^34^, when adjusted for the slightly different assumptions used there.

**Figure 4:**
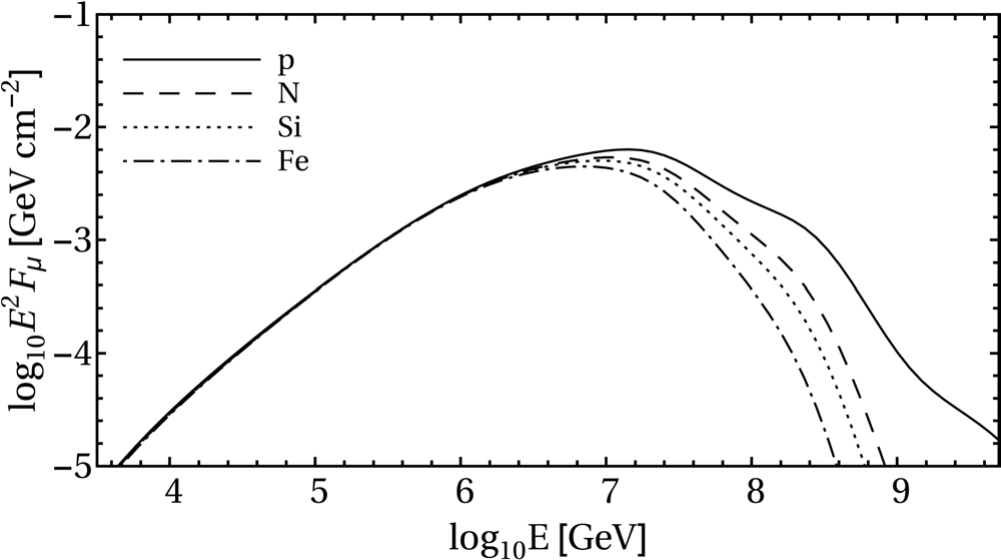
Fluence of ${\nu }_{\mu }+{\bar{\nu }}_{\mu }$ for a single TDE and different (pure) injection compositions. The chosen parameters are *z* = 0.01, *L*_*X*_ = 47.5ergs^−1^, *R* = 10^9.8^km, and ${\xi }_{A}=10$, as used in^33^ for the proton injection case (solid curve in this plot).

In Fig. 4 we illustrate the dependence of the neutrino fluence of a single TDE on the (pure) injected nuclear composition. Spectra for different nuclear species–for the same injection luminosity–are shown. The case of proton composition matches the corresponding one in ref.^33^. It is evident that the change of composition affects the fluence mostly beyond its peak, at $E\gtrsim {10}^{7}\,{\rm{G}}{\rm{e}}{\rm{V}}$, as was already found in^38^.

In the current paper we discuss the C-O white dwarfs as possible origin of UHECRs and neutrinos, simulated by the most abundant isotope of nitrogen, ^14^N as representative for the most abundant isotope of carbon, ^12^C, and oxygen, ^16^O. Differences can be expected relative to a more realistic mixed C-O injection, both in the source and in the extragalactic propagation; some are due to the fact that for both ^12^C and ^16^O, the *α*-particle ejection is relevant, which could result in a slightly more efficient disintegration. The lack of cross section measurements for this channel^37,57^ contributes to the uncertainties on the predictions of UHECR observables.

The propagation of the UHECRs between the source and Earth is modeled with the *SimProp* code^41^, which takes into account nuclei photo-disintegration and photo-meson production, as well as the energy losses due to electron-positron pair production and to the redshift of energy. Simulations including the Puget-Stecker-Bredekamp (PSB) model^82^ for photo-disintegration are used, while for the photo-meson production the cross section for single-pion production is employed. For the extragalactic background photon field, we follow the model in^83^. For UHECRs the horizon is limited to redshift $z\sim 1$; however, since we want to study also the neutrinos produced during the propagation we use simulations computed up to *z* = 6. A detailed discussion about the interface between the *NeuCosmA* code and the computation of the final observables through *SimProp* is given in^38^.

The UHECR spectrum and composition measured by the Pierre Auger Observatory^36,47^ are fitted above 10^19^ eV, with the same technique used in^38^. A penalty for the overshooting of the flux at the lowest energies is included in the fit.

## Electronic supplementary material

Supplementary Material
