# Supplementary material for: Tidally disrupted stars as a possible origin of both cosmic rays and neutrinos at the highest energies
Source: Sci Rep. 2018 Jul 17;8:10828. doi: 10.1038/s41598-018-29022-4 (PMC6050258; doi:10.1038/s41598-018-29022-4)
Supplement: Supplementary file 1 — Supplementary Material [file 41598_2018_29022_MOESM1_ESM.pdf]

SUPPLEMENTARY MATERIAL
Tidally disrupted stars as a possible origin of both cosmic rays and neutrinos at the highest energies

Daniel Biehl[1], Denise Boncioli[1], Cecilia Lunardini[2], Walter Winter[1]
[1]*Deutsches Elektronen-Synchrotron (DESY), Platanenallee 6, D-15738 Zeuthen, Germany*
[2]*Department of Physics, Arizona State University, 450 E. Tyler Mall, Tempe, AZ 85287-1504 USA*

(Dated: June 27, 2018)

I Alternative fit scenarios and cosmogenic neutrinos

As a further illustration of the fit results given in the main text of the paper, Suppl. Fig. 1 shows the same observables as in Fig. 1, for the three parameter sets marked in Fig. 2 as A (same as in Fig. 1), B (corresponding to the parameters which best describe the PeV neutrino data points), and C. It appears that for cases B and C one of the two data sets (UHECR or neutrino) cannot be described, therefore also a good joint description cannot be found.

Let us discuss the effect of systematic uncertainties on the UHECR fit. To study the effect of uncertainties on UHECR propagation, we generated results with alternative cross sections used in the photo-disintegration of nuclei, specifically the TALYS [1] cross sections for nuclei with $A \geq 12$ and cross sections as in [2] for $A < 12$. We find that the UHECR best fit point corresponds to lower photon density, and is found within the 5σ region in Fig. 2 (main text), which can therefore be considered as a robust estimate of the allowed parameter space. A similar change in the position of the best fit is expected if an alternative model is used for the extragalactic background light, such as the one in [3]. It has a higher peak flux in the far infrared with respect to [4], which implies an increase of the photo-disintegration efficiency in a similar way as using a higher number of channels in the cross section model (as already pointed out in [5, 6]).

In the main text of the paper we fix the spectral index of the primary injected nuclei to two. Although this choice is motivated by Fermi acceleration, we also tested the impact of harder or softer spectra on our result reported in Fig. 1. Assuming a softer spectrum at injection, the emissivity of the sources is higher, with the consequence that the corresponding neutrino production in the source increases. The reason for that is that the ratio between the high energy flux at the source, which is the one that matters in the comparison to the CR spectrum data after propagation, to the accelerated spectrum in the whole energy range becomes smaller. Moreover, the transition from light to heavy composition happens at lower energies than in the case with spectral index equal to two. On the contrary, a harder injection spectrum hardly affects our main result; only the transition from light to heavy composition is sharper. This considerations are based on the parameters for the combined description of the cosmic ray and neutrino data. Of course, the parameters of the best fit change when considering harder or softer spectral indices at the source, with the interesting consequence that with a softer spectral index the best fit region is shifted towards higher cutoff energies at the injection, meaning to the upper right region of the parameter space in Fig.2 of the main text.

To account for the uncertain evolution with redshift of the TDEs rate, we repeated the calculations using a phenomenological rate, $\tilde{R}(z) \propto (1+z)^m$, with $m \geq 0$. Since the UHECR spectrum is mostly sensitive to the local universe, it can be reasonably reproduced even with $m = 0$ or 1, requiring a higher baryonic loading with respect to what found in the negative source evolution case. For $m \gtrsim 2$, the luminosity required to fit the UHECR data results in overproducing the neutrino flux, and a good joint description of UHECRs and neutrinos is impossible.

As an additional prediction of our model, we show the expected cosmogenic neutrino flux (for one flavor) in Suppl. Fig. 2 for the same parameters as in Suppl. Fig. 1. The flux is well below the sensitivity of the GRAND experiment [7] (see figure). The suppression, compared to other predictions in the literature, is mostly due to the negative redshift evolution of the sources. An order of magnitude enhancement, reaching the design sensitivity of GRAND, is found

---

Correspondence should be addressed to daniel.biehl@desy.de (Daniel Biehl), denise.boncioli@desy.de (Denise Boncioli), cecilia.lunardini@asu.edu (Cecilia Lunardini), walter.winter@desy.de (Walter Winter)

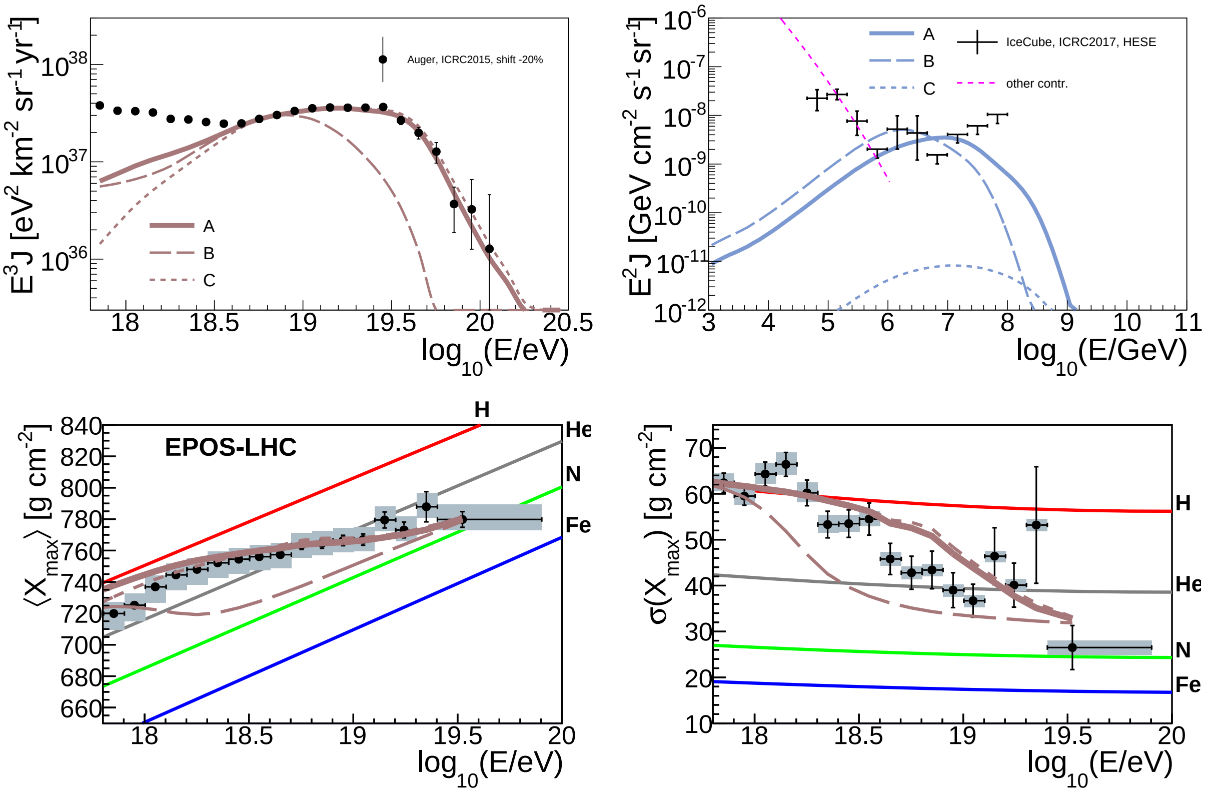

Supplementary Figure 1: Cosmic ray and (muon) neutrino observables obtained with the parameters corresponding to points A, B and C in Fig. 2 (main text).

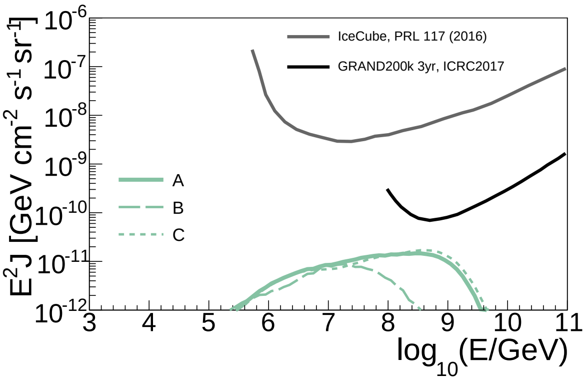

Supplementary Figure 2: Cosmogenic (muon) neutrino flux obtained with the parameters corresponding to points A, B and C in Fig. 2 (main text).

for the alternative model with positive evolution, $m \simeq 2$. This implies, however, that the PeV neutrino data points are overshot, as pointed out previously.

We also study here the contribution to the cosmic-ray and neutrino fluxes as a function of the redshift of the sources in Suppl. Fig. 3. The cosmic-ray horizon, due to the interactions of UHECRs with the extragalactic background light, strongly limits the portion of the universe contributing to the cosmic-ray flux. Although the fraction of the cosmic rays contributing to the total cosmic-ray flux depends somewhat on the parameters at the source, more than 90% of the flux above $10^{17.8}$ eV comes from $z \lesssim 0.25$ if we consider a negative source evolution and the parameters used in Fig. 1 in the main text, and within $z \lesssim 0.50$ if we consider a flat source evolution. For the neutrino (and also diffuse X-ray) flux, the sensitivity to the source evolution is much stronger with respect to the cosmic rays (as one

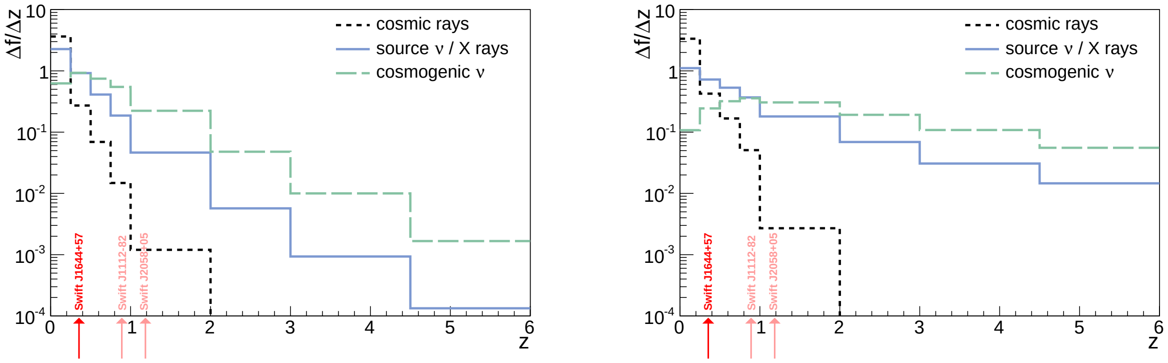

Supplementary Figure 3: Fraction of cosmic rays, source neutrinos and cosmogenic neutrinos per redshift bin, corresponding to the negative source evolution used in this work (left panel) and to a flat source evolution (right panel). The redshifts of the jetted TDEs, according to the classification given in [8] and used also in [9], are marked with arrows. The parameters chosen in this work are motivated by the observation of Swift J1644+57. Note that $\sum_i (\Delta f/\Delta z)_i \, \Delta z_i = 1$ is normalized when summed over all bins i, taking into account the variable bin widths Δz_i.

can see comparing the two panels in Suppl. Fig. 3), and the the redshift dependence of the fractional contributions is very different. What is interesting to stress here is that the fraction of the neutrinos produced in the source and the ones produced in the propagation of the cosmic rays per redshift interval have different behaviors, as shown in Suppl. Fig. 3. This is due to the fact that the interaction length of the cosmic rays becomes smaller with increasing redshift ($\lambda \propto (1+z)^{-3}$) due to the blueshifted target photons. As a consequence, a major contribution to the cosmogenic neutrinos comes from the distant sources. This difference is larger for a negative source evolution than for a flat evolution, as one can see comparing the left and right panels of Suppl. Fig. 3, and it becomes even smaller in case of a source evolution $\propto (1+z)^2$. It is also relevant to note that the redshift distribution of cosmic rays and neutrinos is similar for negative source evolution, especially in the redshift range of the TDEs shown as references. Therefore, neutrinos from TDEs may indeed be indicative for the origin of cosmic rays, as discussed in the main text, and TDEs may be ideal for multi-messenger searches.

II Comparison to proton injection

We show a set of plots here (Suppl. Fig. 4, Suppl. Fig. 5, and Suppl. Fig. 6) for the case of pure proton injection; while this scenario cannot describe the UHECR composition data, it may be interesting for comparison *e.g.* to Ref. [9].

Ref. [9] demonstrates that the diffuse neutrino flux can be described using a pure proton composition in the TDE; the parameter choice corresponding to the base case scenario discussed in there is marked in Suppl. Fig. 4 by a star. The proton-only fit corresponds to the optically thin (to photo-meson production) case, see Suppl. Fig. 4, right panel – as it was indeed implied in Ref. [9]. However, the parameter choice in Ref. [9] corresponds to a higher luminosity compared to what has been found in the current study at the best-fit. As a consequence, the baryonic loading found in [9] required to power the diffuse neutrino flux implies that the UHECR flux is too high by about a factor of seven (apart from different neutrino data used for reference there). This means that for protons, only about 10-15% of the diffuse neutrino flux can be powered by jetted TDEs (in consistency with what has been found in Refs. [10, 11]). Since the required energy injection rate for UHECR nuclei is higher because of a shorter attenuation length, this problem does not occur for nuclei.

A consequence of the proton composition at the source is the enhancement of the cosmogenic neutrino flux of a factor of ~ 4 with respect to the nitrogen case, as can be seen in Suppl. Fig. 6, resulting in a region within the design sensitivity of GRAND. However, such prediction is probably unrealistic, considering the poor fit of the pure proton scenario to the UHECR data.

III Two-photon annihilation

High-energy gamma rays can be produced e. g. by neutral pion decay as a by-product in photo-hadronic interactions. If photons above TeV energies are able to escape from the source, electromagnetic cascades on the CMB and EBL

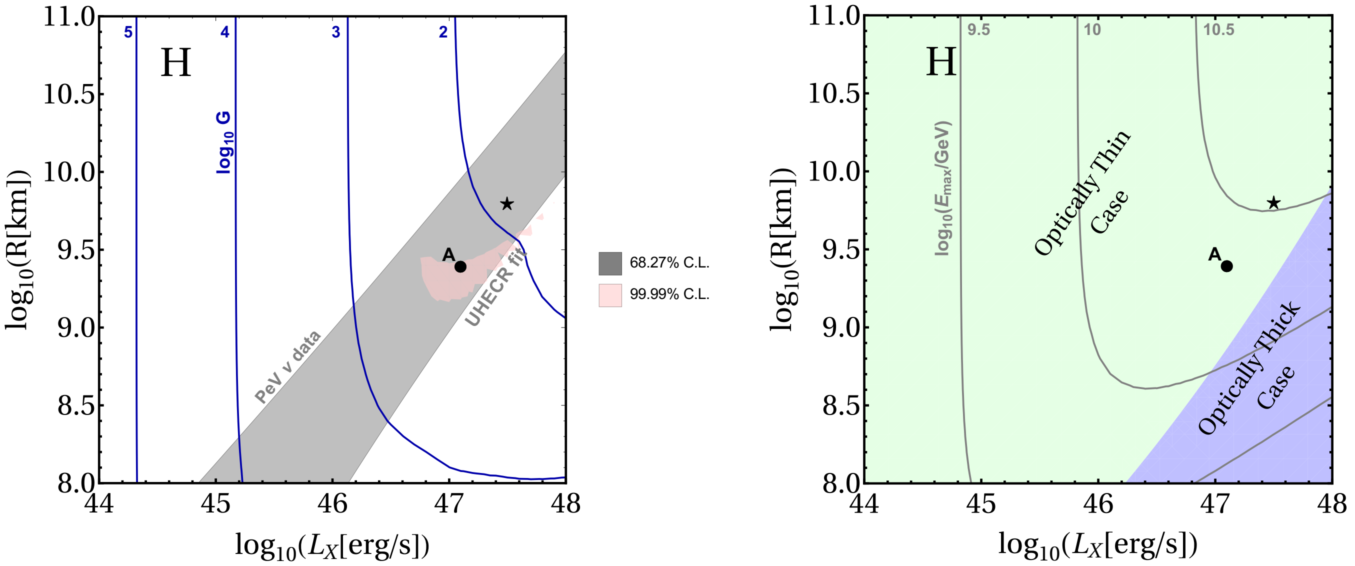

Supplementary Figure 4: Same as in Fig. 2 of the main text, for the case of pure proton injection. Point A refers to the parameters which describes both UHECRs and PeV neutrinos, while the star refers to the standard case discussed in [9] (base case).

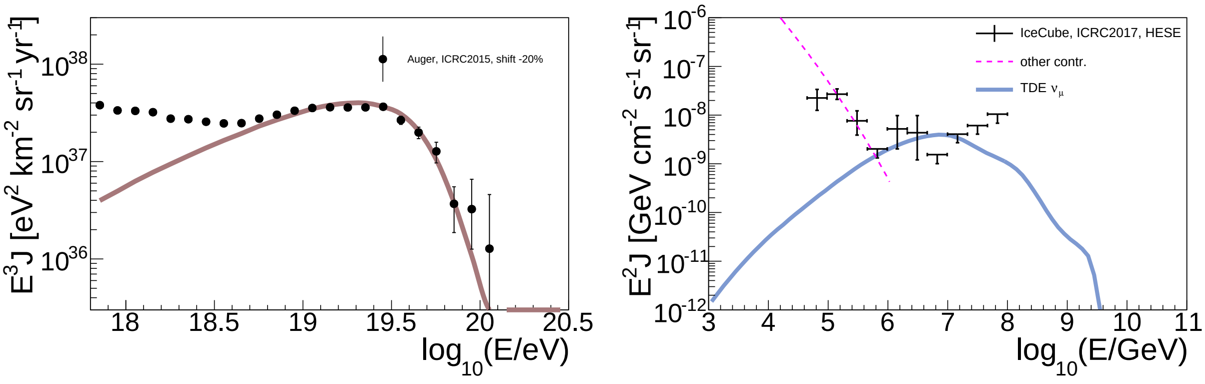

Supplementary Figure 5: Cosmic ray and (muon) neutrino fluxes obtained with the parameters corresponding to the combined fit (point A in Suppl. Fig. 4) with pure proton injection.

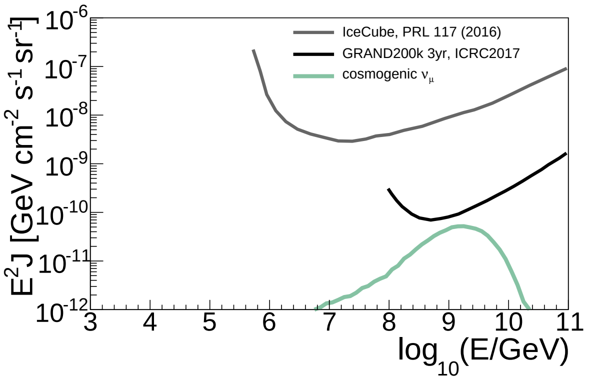

Supplementary Figure 6: Cosmogenic (muon) neutrino flux obtained with the parameters corresponding to the combined fit (point A in Suppl. Fig. 4) with pure proton injection.

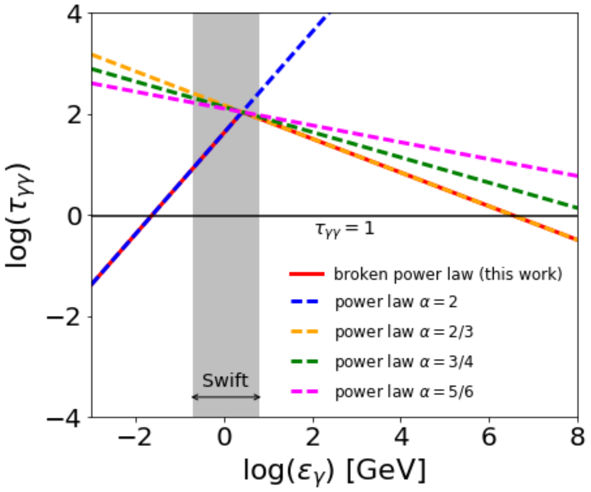

Supplementary Figure 7: Optical depth to two-photon annihilation $\gamma\gamma \rightarrow e^+e^-$ as a function of the gamma ray energy for different spectral indices of the target photon spectrum. The energy band observed for this event, translated into gamma ray energy, is shown by the gray shaded area. The black horizontal line indicates the transition between transparent and opaque.

are initiated, processing high energy gamma rays down to TeV energies. The gamma ray background in this energy range is constrained by *Fermi*, challenging sources which are transparent to gamma rays in this energy range. The situation gets worse for negative source evolution, as most of the gamma rays originate from close by sources, for which the cascades might not even fully develop.

However, $p\gamma$ sources are in general optically thick to high energy gamma rays due to intrasource cascades, *i.e.*, two-photon annihilation may inhibit the direct escape of gamma rays at these energies. For Swift J1644+57, no high energy gamma rays were detected, which is likely due to the opacity to pair production [8, 12]. Since this event was measured in the energy band from 0.4 − 13.5 keV, the extrapolation of the low energy target photon spectrum is important for the behaviour of the optical depth at high gamma ray energies. In this work, we choose a broken power law target photon spectrum $dN_{\rm X}/d\varepsilon'_{\rm X}$ with spectral indices $\alpha_1 = 2/3$ below and $\alpha_2 = 2$ beyond the break energy of $\varepsilon_{\rm X,br} = 1$ keV, which is consistent with the data.

The optical depth for pair creation, $\gamma\gamma \rightarrow e^+e^-$, which is the relevant process for high energy gamma ray escape, can be estimated as done in [13]:

$$\tau_{\gamma\gamma}(\varepsilon_\gamma) \approx 0.1\sigma_{\rm T}\frac{R}{\Gamma}\varepsilon'_{\rm X}\frac{dN_{\rm X}}{d\varepsilon'_{\rm X}}|_{\varepsilon'_{\rm X}=m_e^2c^4/\varepsilon'_\gamma}, \tag{A}$$

where $\sigma_{\rm T} = 6.65 \times 10^{-25}$ cm^2, R is the production radius, ε_γ is the energy of the high energy photon and $\varepsilon_{\rm X}$ is the target photon energy. The corresponding energy in the rest frame of the shock is given by $\varepsilon'_{\rm X} = \varepsilon_{\rm X}/\Gamma$. The factor 0.1 originates from the weighting of different power law segments $\eta(\alpha)$. For the sake of simplicity, we set it to the lowest value relevant for the spectrum used here, which is $\eta(\alpha = 2) \approx 0.12$. Note that for lower α, this weight can be higher [14].

Suppl. Fig. 7 shows the optical depth to two-photon annihilation as a function of the gamma ray energy for different spectral indices, see also discussion in Ref. [13]. Following strictly the broken power law, we find that the source is opaque to gamma ray energies from a few MeV to a few PeV.

At energies of about ~ 10 PeV the source is on the verge of being optically thick. However, the behavior of the target photon spectrum beyond the measurement is unknown. A possible explanation for the non-observation of high energy gamma rays might be the existence of another spectral break or the presence of a thermal component at low energies in the radio range. As there are no constraints by observations, the escape of PeV gamma rays is speculative. We conclude that it is likely that for the sources investigated here, gamma rays are absorbed in the source, and that there is no tension with observations of the gamma ray background.

References

[1] Koning, A. J., Hilaire, S. & Duijvestijn, M. C. TALYS 1.0. In *Proceedings, International Conference on Nuclear Data for Science and Tecnology*, 211–214 (2007).

[2] Boncioli, D., Fedynitch, A. & Winter, W. Nuclear Physics Meets the Sources of the Ultra-High Energy Cosmic Rays. *Scientific Reports* **7**, 4882 (2017).

[3] Dominguez, A. *et al.* Extragalactic Background Light Inferred from AEGIS Galaxy SED-type Fractions. *Mon. Not. Roy. Astron. Soc.* **410**, 2556 (2011).

[4] Gilmore, R. C., Somerville, R. S., Primack, J. R. & Dominguez, A. Semi-analytic modeling of the EBL and consequences for extragalactic gamma-ray spectra. *Mon. Not. Roy. Astron. Soc.* **422**, 3189 (2012).

[5] Alves Batista, R., Boncioli, D., di Matteo, A., van Vliet, A. & Walz, D. Effects of uncertainties in simulations of extragalactic UHECR propagation, using CRPropa and SimProp. *JCAP* **1510**, 063 (2015).

[6] Aab, A. *et al.* Combined fit of spectrum and composition data as measured by the Pierre Auger Observatory. *JCAP* **1704**, 038 (2017).

[7] Fang, K. *et al.* The Giant Radio Array for Neutrino Detection (GRAND): Present and Perspectives. *PoS* **ICRC2017**, 996 (2017).

[8] Burrows, D. N. *et al.* Discovery of the Onset of Rapid Accretion by a Dormant Massive Black Hole. *Nature* **476**, 421 (2011).

[9] Lunardini, C. & Winter, W. High Energy Neutrinos from the Tidal Disruption of Stars. *Phys. Rev.* **D95**, 123001 (2017).

[10] Dai, L. & Fang, K. Can tidal disruption events produce the IceCube neutrinos? *Mon. Not. Roy. Astron. Soc.* **469**, 1354 (2017).

[11] Senno, N., Murase, K. & Meszaros, P. High-energy Neutrino Flares from X-Ray Bright and Dark Tidal Disruption Events. *Astrophys. J.* **838**, 3 (2017).

[12] Aleksić, J. *et al.* Very high energy gamma-ray observation of the peculiar transient event Swift J1644+57 with the MAGIC telescopes and AGILE. *Astron. Astrophys.* **552**, A112 (2013).

[13] Murase, K., Guetta, D. & Ahlers, M. Hidden Cosmic-Ray Accelerators as an Origin of TeV-PeV Cosmic Neutrinos. *Phys. Rev. Lett.* **116**, 071101 (2016).

[14] Svensson, R. Non-thermal pair production in compact X-ray sources: first-order Compton cascades in soft radiation fields. *Mon. Not. Roy. Astron. Soc.* **227**, 403–451 (1987).
